# Editorial: Cancer biomarkers: molecular insights into diagnosis, prognosis, and risk prediction: volume II

**DOI:** 10.3389/fmolb.2026.1999668

**Published:** 2026-09-30

**Authors:** Matteo Becatti, Giuseppe Bronte, Shaoquan Zheng

**Affiliations:** 1 Department of Experimental and Clinical Biomedical Sciences “Mario Serio”, University of Firenze, Firenze, Italy; 2 Department of Translational Medicine, University of Ferrara, Ferrara, Italy; 3 Department of Oncology, University Hospital of Ferrara, Ferrara, Italy; 4 Breast Disease Center, The First Affiliated Hospital, Sun Yat-sen University, Guangzhou, China

**Keywords:** cancer biomakers, liquid biopsy, precision oncology, prognostic biomarkers, risk stratification

The continuing evolution of precision oncology has profoundly changed the concept of a cancer biomarker. Biomarkers are no longer regarded simply as isolated molecular indicators of the presence or absence of disease. Increasingly, they are expected to capture tumor biology, host-tumor interactions, therapeutic vulnerability, and dynamic changes occurring during disease progression and treatment. At the same time, advances in genomics, transcriptomics, molecular imaging, liquid biopsy, and computational analysis are expanding the range of measurable biological signals and creating opportunities to integrate them into clinically meaningful models. The 28 contributions collected in *Cancer Biomarkers: Molecular Insights into Diagnosis, Prognosis, and Risk Prediction: Volume II* illustrate this transition. Spanning different tumor types, biological levels, and methodological approaches, they collectively highlight a shift from single, static markers toward multidimensional and dynamic biomarker strategies capable of supporting diagnosis, risk stratification, prognosis, and treatment monitoring.

A first group of studies emphasizes the importance of linking biomarkers to the molecular mechanisms underlying tumor behavior. In head and neck squamous cell carcinoma, Wang et al. identify RDM1 as a regulator connecting DNA damage response, cell-cycle control, and antioxidant signaling through a newly described RDM1-KEAP1-NRF2 axis. This study exemplifies how mechanistic characterization can transform a candidate biomarker into a potential therapeutic target. A related connection between redox biology and therapeutic response emerges from the work of Ji et al., who implicate TRAP1 in cisplatin resistance in gastric cancer through modulation of oxidative stress, mitochondrial function, DNA damage, and cell death.

Several contributions extend this mechanistic perspective to transcriptomic and multi-gene signatures. Bu et al. combine network analysis with experimental validation to identify CCNA2, CD44, and STAT1 as clinically relevant hub genes in oral squamous cell carcinoma. In lung adenocarcinoma, You et al. highlight ZHX3 as a candidate prognostic biomarker, while Zhuang et al. identify EMCN as a marker associated with vascular-immune crosstalk, illustrating the increasing relevance of the endothelial compartment and tumor microenvironment in biomarker discovery.

Emerging biological processes also provide new frameworks for risk classification. In colorectal cancer, Jiang et al. develop a liquid-liquid phase separation-related gene signature associated with prognosis, immune landscape, and drug sensitivity, while also identifying ARL6IP4 as an experimentally supported component of this biology. In hepatocellular carcinoma, Zhang et al. construct a pyroptosis-related long non-coding RNA signature that links prognosis with immune characteristics and potential immunotherapy stratification. Similarly, Cao et al. develop an anoikis-based risk model for adrenocortical carcinoma and connect its prognostic value with the immune microenvironment. Complementing these original studies, Wang et al. review the emerging concept of disulfidptosis in androgen-related cancers and discuss its potential implications for biomarker development and therapeutic targeting. Together, these studies show that programmed cell death, redox homeostasis, biomolecular condensation, and stromal-immune interactions are becoming integral components of contemporary biomarker research.

Another major theme of this Research Topic is the increasing value of circulating and readily accessible biomarkers. Particularly important is the transition from single measurements to longitudinal monitoring. In metastatic esophageal adenocarcinoma, Du and Fan show that early changes in circulating free DNA during nivolumab-based chemoimmunotherapy are associated with treatment response and survival, illustrating the potential of liquid biopsy to provide information before conventional radiological assessment. In critically ill patients with advanced lung cancer, Tan et al. similarly demonstrate that baseline circulating tumor DNA and its early dynamics add prognostic information for short-term mortality beyond conventional clinical variables. The systematic review and meta-analysis by Li et al. broadens this perspective by showing that multiple circulating biomarkers, particularly inflammatory markers, are associated with short-term mortality in critically ill lung cancer patients.

Dynamic biochemical markers may also capture metabolic aspects of tumor progression and treatment response. Li et al. evaluate baseline and treatment-related changes in LDH and α-HBDH in extensive-stage small-cell lung cancer treated with chemoimmunotherapy, supporting the prognostic relevance of both absolute levels and their trajectories. These observations reinforce an important concept emerging across the Research Topic: for several biomarkers, the direction and magnitude of change during therapy may be more informative than a single pretreatment measurement.

At the same time, conventional circulating biomarkers remain clinically valuable when appropriately integrated with pathological and clinical information. Shen and Kang examine SCC-Ag, CEA, and CA125 in cervical cancer, relating their preoperative levels to pathological characteristics and recurrence. In papillary thyroid carcinoma, Lin et al. show that serum thyroglobulin and anti-thyroglobulin antibodies contribute to prediction of central compartment lymph-node metastasis when integrated with clinicopathological variables. Likewise, Li et al. investigate circulating LILRB2 and APE1 in colorectal cancer and associate elevated levels with pathological characteristics and poorer prognosis.

A particularly strong message across the Research Topic is that combinations of simple biomarkers may outperform individual parameters. In stage I-III colorectal cancer, Wang et al. develop an immune-nutritional prognostic ratio integrating inflammatory, immune, and nutritional information and demonstrate its potential for postoperative risk stratification. In early-stage cervical cancer, Abudoukade et al. similarly show that combining the systemic immune-inflammation index with the prognostic nutritional index provides additional information on postoperative recurrence risk. In gastric cancer, Yuan and Wang propose the preoperative albumin-to-carcinoembryonic antigen ratio as a readily accessible prognostic parameter and incorporate it into a risk-stratification model. The review by Zhang and Liu places these findings in a broader context by examining the CALLY index, which integrates C-reactive protein, albumin, and lymphocyte measurements as a marker of the interconnected inflammatory, nutritional, and immune status of patients with cancer.

Systemic metabolism and oxidative modifications provide additional dimensions of cancer risk and characterization. Using a large prospective cohort, He et al. report an inverse association between plasma histidine and colorectal cancer risk and identify systemic inflammation as a partial mediator of this relationship, linking metabolic and inflammatory biomarkers to cancer susceptibility. In patients with adrenal masses, Choromańska et al. investigate protein oxidation, glycation, carbamylation, and thiol-disulfide homeostasis, suggesting that systemic protein modifications may provide information about adrenal tumor biology and size.

The Research Topic also highlights how imaging can itself become a source of quantitative biomarkers. Ji et al. use intratumoral habitat radiomics derived from dynamic contrast-enhanced MRI to predict axillary response to neoadjuvant chemotherapy in breast cancer, demonstrating the added value obtained by integrating spatial tumor heterogeneity with clinicopathological information. In a conceptually different application of molecular imaging, Kim et al. examine stress-related amygdala metabolic activity measured by ^18^F-FDG PET/CT and its association with distant metastasis in colorectal cancer. This work illustrates an intriguing expansion of cancer biomarker research beyond the tumor itself, toward systemic neuroendocrine and inflammatory processes potentially associated with disease progression.

Genomic technologies provide another route toward increasingly precise tumor characterization. In breast cancer, Liu et al. evaluate HER2 status by next-generation sequencing and compare genomic assessment with established IHC/FISH criteria, while also exploring characteristics of ERBB2 amplification relevant to treatment response. In non-small cell lung cancer, Li et al. demonstrate high concordance in driver-gene detection between bronchial biopsy tissue and matched bronchial brushing cytology, underscoring the clinical importance not only of *which* biomarker is measured, but also of obtaining reliable molecular information from less invasive and more readily available specimens.

An additional direction is represented by integrative molecular models designed for diagnostically or clinically challenging settings. Zhao et al. combine candidate inverse miRNA–mRNA pairs with LASSO-based modeling to distinguish benign from malignant breast lesions, providing a tissue-based molecular adjunct that may be particularly relevant when conventional assessment is uncertain. In a different clinical context, Chen et al. apply transcriptomic and immune-cell profiling to tumor patients admitted to a surgical intensive care unit and derive an immune-related prognostic signature for short-term survival. These approaches demonstrate how computational integration can convert complex molecular datasets into clinically interpretable risk estimates.

Taken together, the contributions to this Research Topic reveal several convergent trends. First, biomarker research is increasingly moving from isolated molecules toward composite signatures that incorporate multiple biological domains. Second, dynamic measurements, including longitudinal cfDNA/ctDNA and metabolic markers, are emerging as particularly informative tools for monitoring disease and therapeutic response. Third, the boundary between tumor biomarkers and host biomarkers is becoming less distinct: nutritional state, systemic inflammation, oxidative stress, immune competence, vascular biology, and even neuroendocrine activity can contribute to clinically relevant risk information. Finally, advanced molecular technologies and relatively inexpensive laboratory measurements should not be considered competing strategies. Rather, they can provide complementary layers of information whose value depends on the clinical question, accessibility, reproducibility, and capacity to alter patient management.

Important challenges nevertheless remain before many of these approaches can be incorporated into routine oncology. External and prospective validation, harmonization of analytical procedures and cutoff values, evaluation across diverse patient populations, and demonstration of incremental clinical utility beyond existing staging and scoring systems are recurrent priorities. Computationally derived signatures additionally require transparent modeling, robust validation, and careful assessment of generalizability, while emerging mechanistic biomarkers need confirmation that biological plausibility translates into actionable clinical information.

The studies gathered in this Research Topic therefore portray cancer biomarkers not as fixed labels, but as increasingly dynamic representations of tumor and host biology. The next step for the field will be to transform this expanding molecular information into standardized, interpretable, and clinically actionable tools. Achieving this goal will require integration rather than accumulation: integration across molecular layers, across time, and ultimately across the biological and clinical variables that determine the course of cancer in each individual patient.

